# Improving weekend handover in a teaching hospital elective general surgery department

**DOI:** 10.3389/fsurg.2023.1263502

**Published:** 2023-10-06

**Authors:** Eloise Dexter, Josephine Walshaw, Ayla Brown, Tehmina Nadeem, Marina Yiasemidou, Terence Lo

**Affiliations:** ^1^ Department of General Surgery, Hull University Teaching Hospitals NHS Trust, Hull, United Kingdom; ^2^ Department of Health Sciences, Faculty of Sciences, University of York, York, United Kingdom; ^3^ Department of General Surgery, The Royal London Hospital, Barts Health NHS Trust, London, United Kingdom

**Keywords:** quality improvement, handover, patient safety, teamwork, general surgery

## Abstract

**Background:**

Effective documentation and transfer of clinical information are vital for the continuity of care, patient safety, and maintaining medico-legal records, as outlined by the Royal College of Surgeons “Safe Handover: Guidance from the Working Time Directive working party”. Our elective surgery weekend team cross-covers both Colorectal and Upper Gastrointestinal surgical specialties across multiple wards, which poses a significant challenge. The aim of this study was to improve the documentation of patients' weekend plans through the introduction of a weekend handover proforma.

**Method:**

We reviewed the weekend plans of 199 patients overall. 41 records were initially reviewed over a 2-week period. The surgical multidisciplinary team was then surveyed to establish the need for an improved weekend handover. Following this, a weekend handover proforma was introduced as part of the Friday ward round and education on the expectations were provided at a local Surgery Clinical Governance meeting. The documentation of the weekend plan was reviewed for 158 patients over a 6-week period and a post-intervention survey was disseminated.

**Results:**

The preliminary survey highlighted concerns for delayed discharges and patient safety over the weekend, with 88.2% of respondents agreeing a weekend handover proforma would be beneficial. The initial data confirmed inadequate documentation of diagnosis (19.5%), operation/procedure (28.1%), and weekend plans for blood tests (19.5%), discharge planning (2.4%), diet (46.3%), antibiotics (19.5%), intravenous (IV) fluids (22.0%), mobility (19.5%) and drain/wound care (37.5%). After education and implementing a weekend handover proforma, these results increased for documentation of diagnosis (61.2%), operation/procedure (83.2%), blood tests (59.7%), and discharge planning (85.8%). However, there was little improvement in diet (53.0%) and no improvement in the weekend plans for antibiotics (14.2%), IV fluids (17.2%), mobility (14.9%) and drain/wound care (20.2%). The post-intervention survey showed an improvement across all areas, notably continuity of care and patient safety, with 95.5% of individuals finding the weekend handover proforma aided in patient care over the weekend.

**Conclusion:**

Education of the ward team and implementation of a weekend handover proforma resulted in a marked improvement in the documentation of patients' weekend plans, which is essential to ensure the continuation of safe and effective patient care.

## Introduction

Effective documentation and transfer of clinical information are vital for ensuring continuity of care, patient safety, and maintaining medico-legal records, as outlined by the Royal College of Surgeons (RCS) “Safe Handover: Guidance from the Working Time Directive working party” (1). The weekend handover, in particular, presents a unique and critical juncture where the transfer of patient information, responsibility, and decision-making must occur smoothly between medical teams. Inadequate handovers can result in delayed or compromised care, leading to adverse patient outcomes, increased length of stay, and heightened healthcare costs (2–4).

Within our hospital's elective Gastrointestinal (GI) Surgery department, there is a lack of a defined method for patient handover from week to weekend doctors. Team-to-team handovers occurred intermittently and were a non-standardised doctor-dependent process. This lack of a routine handover practice resulted in doctors having limited familiarity with patients, potentially compromising patient safety, quality of care, and leading to longer ward rounds. Clinicians often require multisource information (electronic and paper written records) in order to establish the diagnosis and current issues.

Additionally, the elective GI Surgery weekend team operates on staff cross-covering both Colorectal and Upper GI specialties across multiple wards. It is reported that due to the increase in cross-covering specialties doctors may not only be unfamiliar with a specific patient's case but also unfamiliar with the management of their conditions, particularly in a tertiary unit with highly complex specialised surgeries (5).

The standardisation of handover is vital to improve efficacy and patient safety, and can be aided by standardised proformas. Previous studies have shown that implementing a standardised proforma can result in increased weekend discharges, decreased length of stay, surgical decision making and overall patient care (6–10).

The aim of this project is to adopt a multidisciplinary approach to collaboratively identify shortcomings in the current weekend handover in an elective GI surgery department and to improve the documentation of patients' weekend plans through the introduction of a standardised weekend handover proforma. The outcomes from this project will also offer valuable insights for other surgical departments looking to implement similar patient safety initiatives.

## Methods

The Quality Improvement (QI) project was conducted at Castle Hill Hospital, Hull University Teaching Hospitals NHS Trust, UK. This project focused specifically on the Upper GI and Colorectal wards, which house elective and long-stay General Surgical patients within the trust.

### Survey

A preliminary survey was distributed among the multidisciplinary team (MDT) working on both Upper GI and Colorectal surgical wards. This was designed to capture the perspectives of various healthcare professionals, including junior and consultant doctors, nurses, physiotherapists, and pharmacists. The survey consisted of seven questions using a 5-point Likert scale, with respondents rating on a scale: 1 (never), 2 (rarely), 3 (sometimes), 4 (often), and 5 (always). Two open-text questions were also included to provide participants with the opportunity to provide explanations for their ratings and suggest what they thought should be included on a weekend handover proforma. Additionally, respondents were asked if they felt a weekend handover would be beneficial.

Following the implementation of the interventions a second survey was distributed addressing the same seven questions, to evaluate the impact of these changes.

### Baseline data collection

Baseline data was collected over a period of two weeks in October 2022 using a bespoke data collection form. Data was collected by examining the most recent documentation from the Friday ward round to assess the weekend plan, all patients who were inpatients on a Friday were included in the analysis. No patient-identifiable information was gathered. Demographic parameters for each patient included the ward and specialty (Upper GI or Colorectal), diagnosis, and operation/procedure. Further data was collected on whether weekend blood tests, discharge, diet, intravenous (IV) fluids, antibiotics, mobility, and wound/drain care plans were documented. A team consisting of three surgical junior doctors collected the data; the data collection team was kept small and in constant communication in order to standardise what was considered to be “clear” in the notes.

### Interventions

After conducting the questionnaire and gathering baseline data, it became evident that the implementation of a handover process was necessary and favoured by the clinical team. To formulate an effective handover, informal discussions were held with senior colleagues, particularly those who had experience in other trusts utilising paper handover documents already. The information needed for doctors at all levels, as well as important considerations for allied healthcare professionals, were taken into account using the free text answers from the survey and discussions. By incorporating insights from published research on effective handover methods (11–13), a tailored handover proforma was developed to suit the needs of our specific trust (Appendix 1).

The proforma included a section for patient demographics, date, time and senior leading the ward round. Sections for presenting complaint(s), any operation/procedures, and a list of ongoing issues were included. Subsequently, a white space section for the Friday ward round documentation of the weekend plan, and specifically the weekend blood tests and discharge plan, were included. To enhance visibility and accessibility, the handover document was printed on yellow paper, following the example of a successful study (12), and placed within the patient notes at the correct date. A laminated copy of the handover proforma was prominently displayed on the ward, and pre-printed yellow proformas were made readily available in the notes trolley. Additionally, reminders to prepare the handover proformas were regularly disseminated through group messages by registrars on Thursday and Friday mornings to encourage completion of the proformas. This was to be completed for all patients seen on the Friday morning ward round.

The results from the baseline data were analysed and presented at the departmental Clinical Governance Meeting in November 2022. Education was provided to raise awareness about the importance of a safe weekend handover and the weekend handover proforma was introduced. Following these interventions, data was collected over a 6-week period between November and December 2022. Data regarding the weekend plan was collected using the same method as the pre-intervention baseline data collection. A post-intervention survey was also distributed to doctors and allied healthcare professionals working in the department.

The derived findings were then presented during the May 2023 departmental Clinical Governance meeting, serving as a platform for discussion and analysis with the team. This collaborative evaluation aimed to gauge the form's impact and effectiveness within the clinical settings of the ward for all levels. Moreover, ongoing and continuous communication and collaboration existed between both Upper GI and Colorectal teams throughout the process.

### Data analysis

Descriptive statistics were used for the results of the survey and for the analysis of the data collected regarding the documentation of the weekend handover. This was done for both the baseline and the post-intervention variables used; facilitating comparison between both data sets.

This article is reported based on the Revised Standards for Quality Improvement Reporting Excellence (SQUIRE 2.0) guidelines (14).

## Results

### Pre-intervention survey

17 individuals responded to the pre-intervention survey, this included junior doctors of all levels, consultants, a pharmacist, nurse and physiotherapist (Table 1). Based on the most popular response to each survey question, 64.7% of individuals were “sometimes” satisfied with the level of information documented in the notes regarding the weekend plan. 76.5% of respondents state that tasks “often” took longer over the weekend due to the information contained in the notes. Additionally, 52.9% of participants “often” encountered difficulty in identifying weekend tasks from the notes. 58.8% of respondents felt important jobs were “rarely” accomplished over the weekend, discharges were “often” delayed over the weekend, and there was “often” a lack of continuity of care due to the information in the notes. Notably, 47.1% of respondents were “often” concerned about patient safety over the weekend due to the information in the notes. Consequently, 88.2% of individuals felt the introduction of a weekend handover proforma would contribute to improved patient care and facilitate discharges.

**Table 1:** Pre-implementation survey results.

	Never	Rarely	Sometimes	Often	Always
Satisfaction with **level of information** in notes	5.90%	29.40%	**64**.**70%**	0%	0%
**Jobs take longer** over the weekend due to the information in the notes	0%	0%	11.80%	**76**.**50%**	11.80%
Difficulty **identifying jobs** for the weekend due to information in the notes	0%	11.80%	35.30%	**52**.**90%**	0%
Satisfaction that all important **jobs get carried out** over the weekend	0%	**58**.**80%**	29.40%	11.80%	0%
**Discharges are delayed** over the weekend due to information in the notes	0%	5.90%	23.50%	**58**.**80%**	11.80%
**Lack of continuity of care** due to information in note	0%	0%	35.30%	**58**.**80%**	5.90%
**Worry about patient safety** over the weekend due to information in notes	0%	17.60%	35.30%	**47**.**10%**	0%

The most frequent answer for each question is in bold.

Analysis of the free-text survey responses highlighted several key issues. Firstly, there was little to no handover between specialties from the week, and the weekend plans were often unclear in the notes. Therefore, extensive reading was often required to gather information for the safe continuity of care. Respondents expressed concerns about the lack of progress in plans over the weekend, missed tasks, and delayed assessment of deteriorating patients. In terms of desired inclusion on the handover proforma, the majority of respondents emphasised the importance of clearly stating the diagnosis, procedure, current issues, and plan, including any required blood tests or anticipated discharges for the weekend. Additionally, a variety of additional suggestions were made including antibiotics, fluids, drain removal, mobility, chest physiotherapy, and escalation status documentation.

### Weekend handover documentation—pre-intervention

Pre-intervention data was collected for 41 patients, of which 32 patients had operations/procedures. The diagnosis was documented in 8/41 (19.5%) of the cases. Of those who underwent operations/procedures, only 9/32 (28.1%) had clearly documented information regarding this. Furthermore, the documentation pertaining to the need for blood tests to be taken over the weekend was present for 8/41 (19.5%). A discharge plan was documented 1/41 (2.4%) of the patients. IV fluids were documented in 9/41 cases (22.0%), antibiotics and mobility in 8/41 cases (19.5%), and diet in 19/41 cases (46.3%). Wound/drain information in patients who underwent operations/procedures was documented in 12/32 (37.5%) of cases.

### Weekend handover documentation—post-intervention

Following department education and the implementation of the handover proforma, a total of 158 patients were included in data analysis (Table 2). Of those, 134 used the proforma and 24 did not. During this period 141 patients underwent operations/procedures, of those 119 used the proforma and 22 did not.

**Table 2:** Pre- and post-implementation of the weekend handover proforma data.

		UGI	Colorectal	Both
Is the diagnosis clear	Pre proforma	3/15 (20%)	5/26 (19.2%)	8/41 (19.5%)
	Proforma used	43/56 (76.8%)	39/78 (50%)	82/134 (61.2%)
	Proforma not used	4/15 (26.7%)	4/9 (44.4%)	8/24 (33.3%)
Is the operation clear	Pre proforma	4/11 (36.4%)	5/21 (23.8%)	9/32 (28.1%)
	Proforma used	37/45 (82.2%)	62/74 (83.8%)	99/119 (83.2%)
	Proforma not used	4/13 (30.1%)	5/9 (55.6%)	9/22 (40.9%)
Bloods	Pre proforma	7/15 (46.7%)	1/26 (3.8%)	8/41 (19.5%)
	Proforma used	42/56 (75%)	38/78 (48.7%)	80/134 (59.7%)
	Proforma not used	2/15 (13.3%)	2/9 (22.2%)	4/24 (16.7%)
Discharge plan	Pre proforma	1/15 (6.7%)	0/26 (0%)	1/41 (2.4%)
	Proforma used	51/56 (91.1%)	64/78 (82.1%)	115/134 (85.8%)
	Proforma not used	7/15 (46.7%)	4/9 (44.4%)	11/24 (45.8%)
Diet	Pre proforma	11/15 (73.3%)	8/26 (30.8%)	19/41 (46.3%)
	Proforma used	23/56 (41.1%)	38/78 (48.7%)	71/134 (53.0%)
	Proforma not used	5/15 (33.3%)	2/9 (22.2%)	7/24 (29.2%)
IV fluid	Pre proforma	3/15 (20%)	6/26 (23.1%)	9/41 (22.0%)
	Proforma used	8/56 (14.3%)	15/78 (19.2%)	23/134 (17.2%)
	Proforma not used	0/15 (0%)	0/9 (0%)	0/24 (0%)
Antibiotics	Pre proforma	5/15 (33.3%)	3/26 (11.5%)	8/41 (19.5%)
	Proforma used	12/56 (21.4%)	7/78 (9.0%)	19/134 (14.2%)
	Proforma not used	1/15 (6.7%)	0/9 (0%)	1/24 (4.2%)
Mobility	Pre proforma	5/15 (33.3%)	3/26 (11.5)	8/41 (19.5%)
	Proforma used	7/56 (12.5%)	13/78 (16.7%)	20/134 (14.9%)
	Proforma not used	1/15 (6.7%)	0/9 (0%)	1/24 (4.2%)
Wound/drain	Pre proforma	4/11 (36.4%)	8/21 (38.1%)	12/32 (37.5%)
	Proforma used	14/45 (31.1%)	10/74 (13.5%)	24/119 (20.2%)
	Proforma not used	3/13 (23.1%)	0/9 (0%)	3/22 (13.6%)

The most significant improvement was seen in the documentation of weekend discharge plans. Prior to the intervention, only 1/41 cases (2.4%) had a clearly documented discharge plan. Post-intervention this increased to 115/134 cases (85.8%) when the handover proforma was used, compared to 11/24 cases (45.8%) when it wasn't.

Prior to the intervention, the documentation of diagnosis was observed in only 8/41 cases (19.5%). After the intervention, this increased to 82/134 cases (61.2%) when the handover proforma was used, compared to 8/24 cases (33.3%) when it wasn't. Similarly, documentation of a procedure/operation improved from 9/32 cases (28.1%) pre-intervention, to 99/119 cases (83.2%) when the handover proforma was used, compared to 9/22 cases (40.9%) when it wasn't. Weekend blood test requirements also showed improvement, increasing from 8/41 cases (19.5%) before intervention to 80/134 cases (59.7%) when the handover proforma was used, compared to 4/24 cases (16.7%) when it wasn't.

However, there were some areas where only minor improvement or a decrease in the documentation of the weekend plan was observed. Regarding dietary information, there was minor improvement from 19/41 cases (46.3%) pre-intervention, to 71/134 cases (53.0%) when the handover proforma was used, compared to 7/24 cases (29.2%) when it wasn't. IV fluid requirement documentation decreased from 9/41 (22.0%) pre-intervention, to 23/134 (17.2%) when the handover proforma was used, compared to 0/24 (0.0%) when it wasn't. Antibiotic documentation went from 8/41 (19.5%) pre-intervention, to 19/134 (14.2%) when the handover proforma was used, compared to 1/24 (4.2%) when it wasn't. Documentation of mobility decreased from 8/41 (19.5%) pre-intervention, to 20/134 (14.9%) when the handover proforma was used, compared to 1/24 (4.2%) when it wasn't. Finally wound/drain care documentation decreased from 12/32 (37.5%) pre-intervention in patients who underwent operations/procedures, to 24/119 (20.2%) when the handover proforma was used, compared to 3/22 (13.6%) when it wasn't.

### Post-intervention survey

22 individuals responded to the post-intervention survey, including junior doctors of all levels, consultants, and nurses (Table 3). Based on the most popular response to each survey question, 77.3% of individuals were “often” satisfied with the level of documentation in the notes regarding the weekend plan. 50% of individuals state the weekend handover proforma “often” reduces the length of time jobs take over the weekend, 40.9% found it “always” easier to identify weekend jobs, and 45.5% felt the important jobs were “often” completed over the weekend. 36.4% of individuals felt that discharges were “sometimes” delayed over the weekend. Importantly, respondents felt that there was “rarely” (36.4%) or “never” (27.3%) a lack in the continuity of care over the weekend, and an overwhelming majority felt there was “rarely” (40.9%) or “never” (36.4%) a concern about patient safety over the weekend since the introduction of the handover proforma. Consequently, 95.5% of individuals found the introduction of a weekend handover proforma aided in patient care over the weekend.

**Table 3:** Post-implementation survey results.

	Never	Rarely	Sometimes	Often	Always
Satisfaction with **level of information** in notes	0%	0%	4.5%	**77**.**3%**	18.2%
Weekend handover proforma aids to **reduce the length of time that jobs take** over the weekend	9.1%	0%	18.2%	**50%**	22.7%
Weekend handover proforma aids to **identify jobs over** the weekend?	0%	4.5%	18.2%	36.4%	**40**.**9%**
Satisfaction that all important **jobs get carried out** over the weekend	0%	9.1%	22.7%	**45**.**5%**	22.7%
**Discharges are delayed** over the weekend due to lack of information in the notes	9.1%	31.8%	**36**.**4%**	18.2%	4.5%
**Lack of continuity of care** due to information in note	27.3%	**36**.**4%**	13.6%	18.2%	4.5%
**Worry about patient safety** over the weekend due to information in notes	36.4%	**40**.**9%**	9.1%	13.6%	0%

The most frequent answer for each question is in bold.

Analysis of the free-text survey responses were overwhelmingly positive. Responses state that the addition of the proforma had made a big difference to aid in weekend ward work, help patients progress more after surgery, and provide a much clearer and easier way to pick out jobs needed over the weekend, particularly when caring for patients the team are unfamiliar with. However, an issue highlighted was that occasionally the proforma was not completed for all patients, particularly patients who had operations on Friday and were not seen as part of the Friday ward round.

Further suggested changes to future versions of the proforma were also encouraged. These included dietary requirements including total parenteral nutrition, antibiotic plans, and patient-specific escalation plans for potential post-operative complications.

## Discussion

This QI project demonstrates the positive impact of implementing a weekend handover proforma, accompanied by departmental education on the completeness of documentation during the weekend handover process. By ensuring clear documentation before the weekend, it promotes more efficient ward rounds, facilitates better job identification, and enhances patient safety (1, 3, 15).

The initial implementation of the handover proforma involved active participation and collaboration among various healthcare professionals, including junior doctors, consultants, nurses, matrons, and ward clerks. This collaborative approach ensured the sustainability of the project despite the rotational nature of doctors across different levels. To facilitate widespread adoption, the handover proforma was designed to be user-friendly and require minimal guidance for completion, considering the frequent rotation of doctors. Special attention was given to ensuring the handover proforma's clarity and comprehensibility. It was important to introduce something that would assist rather than burden the doctors completing the proforma, as excessive workload has been shown to be a significant barrier to implementation success (12, 16). Although completing the proforma initially may increase workload, it served as a valuable reference throughout the week, simplifying subsequent weekend handover proforma completion and potentially resulting time savings by reducing information searching for referrals and discharge letters. To ensure ongoing adherence and effectiveness, the inclusion of the weekend handover proforma is being implemented into the surgical department induction pack.

The project yielded highly positive results, demonstrating significant improvements in the documentation of crucial areas such as diagnosis, operation/procedure, and weekend plans for discharge and blood tests. These areas were identified as key improvement targets based on feedback from the pre-intervention survey. The handover proforma enabled the weekend team to access vital information and current issues without extensive note-searching, reducing the risk of overlooking critical issues and enabling timely decision-making during weekend ward rounds.

A decrease in documentation was observed in areas such as mobility, wound/drain care, IV fluids, and antibiotic plans. As anticipated, these areas did not have dedicated sections in the handover proforma. This decision was made when designing the proforma to avoid overwhelming junior doctors, however a free-text section was included with the assumption that such information would be recorded in the free-text section. The observed decrease in documentation in these areas suggests that the reliance on the free-text section might not have been as effective as anticipated. It is plausible that the absence of these dedicated sections contributed to the reduction in documentation, as some healthcare professionals may have overlooked or considered them less prioritised amidst the busy clinical setting. Therefore, addressing this issue could be crucial in future handover proforma designs.

Furthermore, we observed instances where the handover proforma was not consistently utilised, especially in cases where patients were supposed to be discharged on Friday but had to stay over the weekend due to unforeseen circumstances. In these cases, further education about the completion of the proforma for all patients would be beneficial. Interestingly, even in cases where the handover proforma was not used, improvements were observed in post-intervention areas such as diagnosis, operation/procedure, and discharge planning documentation. This improvement could be attributed to the education provided during the Clinical Governance meeting. However, in all instances where both the handover proforma and education were used together, documentation improved significantly compared to education alone. These findings emphasise the value of combining education alongside the handover proforma to promote a more effective approach to improving weekend handover.

Our data collection followed a standardised and objective assessment approach, similar to other QI projects (13). In addition to the objective assessment of documentation of the weekend plan alone, we recognised the importance of capturing subjective measures from the department in the form of the pre- and post-intervention surveys. By analysing the feedback obtained through the surveys we gained valuable insights, allowing us to tailor our handover proforma based on the departmental needs and obtain a more comprehensive understanding of the impact of the intervention. The results of the post-intervention survey were overwhelmingly positive and showed an improvement across all areas, notably continuity of care and concerns with patient safety over the weekend.

This study acknowledges certain limitations. Our pre- and post-intervention surveys primarily received responses from doctors and nurses with limited participation from the other members of the ward MDT. Consequently, the survey may not fully capture the perspectives of the entire ward team and may introduce a potential bias, restricting the generalisability of the results. Informal discussions revealed that a significant number of physiotherapists and pharmacists within the team had not yet worked on the weekends in the elective GI Surgery department. As a result, their limited experience in the weekend operations hindered their ability to provide accurate survey responses, leading to their decision not to participate in the survey. In future studies, it would be valuable to devise methods that encourage and facilitate the inclusion of a broader range of MDT members who have experience working weekends, in order to obtain a more comprehensive perspective. Future work could also include a comprehensive analysis of the impact of improved weekend handover documentation on patient outcomes, such as reduced length of stay and potential cost savings, to further establish the benefits of the introduced interventions.

Additionally, the surveys were distributed to individuals who were working on the ward during each data collection period. As a result of the rotational nature of doctors and other members of the MDT, the responders to the survey were not necessarily the same individuals in the pre- and post-intervention phases. Instead, it represented a subset of the team working on the wards during those respective periods. While it would be ideal to have the same cohort for both pre- and post-intervention assessments, in practice achieving this poses significant challenges due to the dynamic nature of healthcare teams and their schedules.

## Conclusion

The education of the ward team and implementation of a weekend handover proforma resulted in a marked improvement in the documentation of patients' weekend plans, ensuring the continuity of safe and effective patient care. Despite the rotational nature of doctors, the project has demonstrated sustainability and gained support from various members of the GI Surgery MDT. Ongoing advocacy, regular audits, and further teaching sessions are required to further enhance the effectiveness of the handover proforma.

## Data Availability

The raw data supporting the conclusions of this article will be made available by the authors, without undue reservation.
